# Induced necroptosis limits *Toxoplasma gondii* replication in a RIPK3/MLKL-dependent manner

**DOI:** 10.1128/iai.00479-25

**Published:** 2025-10-07

**Authors:** Billy J. Erazo, Laura J. Knoll

**Affiliations:** 1 Department of Medical Microbiology and Immunology, University of Wisconsin-Madison 5228, Madison, Wisconsin, USA; University of California Davis, Davis, California, USA

**Keywords:** *Toxoplasma gondii*, necroptosis, apoptosis, programmed cell death

## Abstract

*Toxoplasma gondii* is an obligate intracellular parasite capable of subverting host defenses to establish infection. Necroptosis, a lytic pro-inflammatory form of programed cell death, has emerged as a host defense mechanism against intracellular pathogens. However, its relevance in controlling *T. gondii* replication remains unclear. Here, we investigated the role of necroptosis in limiting *T. gondii* replication using bone marrow-derived macrophages (BMDMs) deficient in key necroptotic mediators, RIPK3 and MLKL. We demonstrate that under naïve conditions, *T. gondii* replication proceeds unimpeded in RIPK3^−/−^ and MLKL^−/−^ BMDMs. However, co-treatment with TNF-α and the pan-caspase inhibitor Z-VAD-FMK, conditions that promote necroptosis, significantly reduced parasite replication in wild-type BMDMs but not in those lacking RIPK3 or MLKL. This suppression was dependent on RIPK1 activity, as pharmacological inhibition with Necrostatin-1 abrogated the effect. We further confirmed that TNF-α and Z-VAD-FMK treatment induced necroptotic cell death characterized by loss of plasma membrane integrity, both of which were absent in RIPK3^−/−^ and MLKL^−/−^ cells. These findings establish that the activation of necroptosis can effectively limit *T. gondii* replication in BMDMs and underscore the importance of RIPK1-RIPK3-MLKL signaling in mounting a cell-intrinsic immune defense. Our study provides new insight into the functional capacity of necroptosis in restricting intracellular parasites and highlights its potential as a therapeutic target in toxoplasmosis.

## INTRODUCTION

The parasite *Toxoplasma gondii* is an obligate intracellular protozoan parasite that causes the potentially life-threatening disease toxoplasmosis. This highly adaptable protozoan parasite can infect nearly all nucleated cells and has a broad host range, infecting all warm-blooded animals, including humans (1, 2). In the United States alone, more than 40 million individuals are chronically infected with *T. gondii*, making toxoplasmosis a top-five foodborne illness that results in hospitalization and death (3). Despite its asymptomatic presentation in most immunocompetent individuals, toxoplasmosis can cause severe complications in immunocompromised patients and congenitally infected fetuses.

*T. gondii* is classified into three major clonal lineages, distinguished by differences in virulence and genetic diversity (4). Type I strains (e.g., RH, CAST, and GT-1) are highly virulent, exhibiting rapid replication and acute lethality in murine models (5). Type II strains (e.g., ME49, HART, and WIL) demonstrate moderate virulence and are the most commonly associated with human infections, including chronic, asymptomatic cases and reactivation in immunocompromised individuals (5). Type III strains (e.g., VEG, SOU, and CTG) are considered avirulent in laboratory mice and are less frequently implicated in human disease (5). Consequently, distinct immune responses are elicited depending on the strain, resulting in varied clinical manifestations that range from asymptomatic infections to severe toxoplasmosis in immunocompromised hosts.

The host immune system plays a crucial role in controlling *T. gondii* during infection, where a delicate balance between immune activation and regulation is necessary to limit parasite burden while preventing immunopathology (6, 7). A key tool that the host uses during the immune response is the induction of cell death mechanisms. These mechanisms are vital as they eliminate the intracellular replicative niche of pathogens and regulate inflammation (8). Among these, programed cell death (PCD) represents a highly regulated process distinct from passive or accidental cell death, such as necrosis. PCD is initiated by specific intracellular signaling cascades rather than being a mere consequence of cellular damage (9). The two best-characterized PCD pathways are apoptosis and necroptosis. Apoptosis is a tightly regulated, non-inflammatory process that involves the controlled dismantling of cells, preventing the release of intracellular contents into the surrounding environment and triggering minimal inflammation (9, 10). Conversely, necroptosis is a lytic and pro-inflammatory form of PCD, characterized by cell swelling, plasma membrane rupture, and subsequent release of damage-associated molecular patterns, which amplify immune responses (11–13). Necroptosis can be triggered by receptors on the cell surface, such as tumor necrosis factor receptor 1 (TNFR1) (14). When activated, TNFR1 forms complex I by recruiting RIPK1 in conjunction with other proteins (15, 16). If RIPK1 is deubiquitinated, complex I breaks apart, forming complex II in the cytoplasm (12, 17, 18). This complex includes caspase-8, which initiates apoptosis when active (19). If caspase-8 is blocked, RIPK1 binds to RIPK3, forming the necrosome, which activates MLKL (19, 20). Activated MLKL moves to bind to the cell membrane and disrupt the ion balance (21–24). This causes the membrane to rupture, leading to necroptotic cell death.

*T. gondii* employs multiple strategies to evade host immune defenses, including the inhibition of apoptosis (25). Evidence suggests that *T. gondii* actively manipulates the host apoptotic machinery by inhibiting caspase activation (26, 27). This inhibition occurs at multiple levels of the apoptotic cascade, ensuring host cell survival in a parasite-favorable state. Another strategy to modulate PCD involves using chemical compounds that selectively inhibit or promote specific cell death pathways. For instance, Z-VAD-FMK, a broad-spectrum caspase inhibitor, blocks apoptosis, thereby shifting the balance toward necroptosis under certain conditions (28). Conversely, necroptosis can be pharmacologically inhibited using compounds such as Necrostatin-1 (Nec-1), which targets RIPK1 explicitly by binding to its kinase domain and preventing its autophosphorylation, an essential step in necroptosis initiation (29).

Our laboratory previously demonstrated that RIPK3 plays a role in facilitating host resistance to oral *T. gondii* infection (30). Recent studies have shown that *T. gondii* actively inhibits necroptosis, particularly during its slow-replicating, latent stage (31). However, it remains unclear whether the fast-replicating stage of *T. gondii* is equally capable of evading host necroptosis. Here, we tested the hypothesis that the parasitemia of the rapidly proliferating stage of *T. gondii* can be suppressed through necroptosis without being inhibited by parasite-driven mechanisms.

## RESULTS

### RIPK3 and MLKL absences do not affect *T. gondii* replication in naïve BMDMs

**Fig 1:**
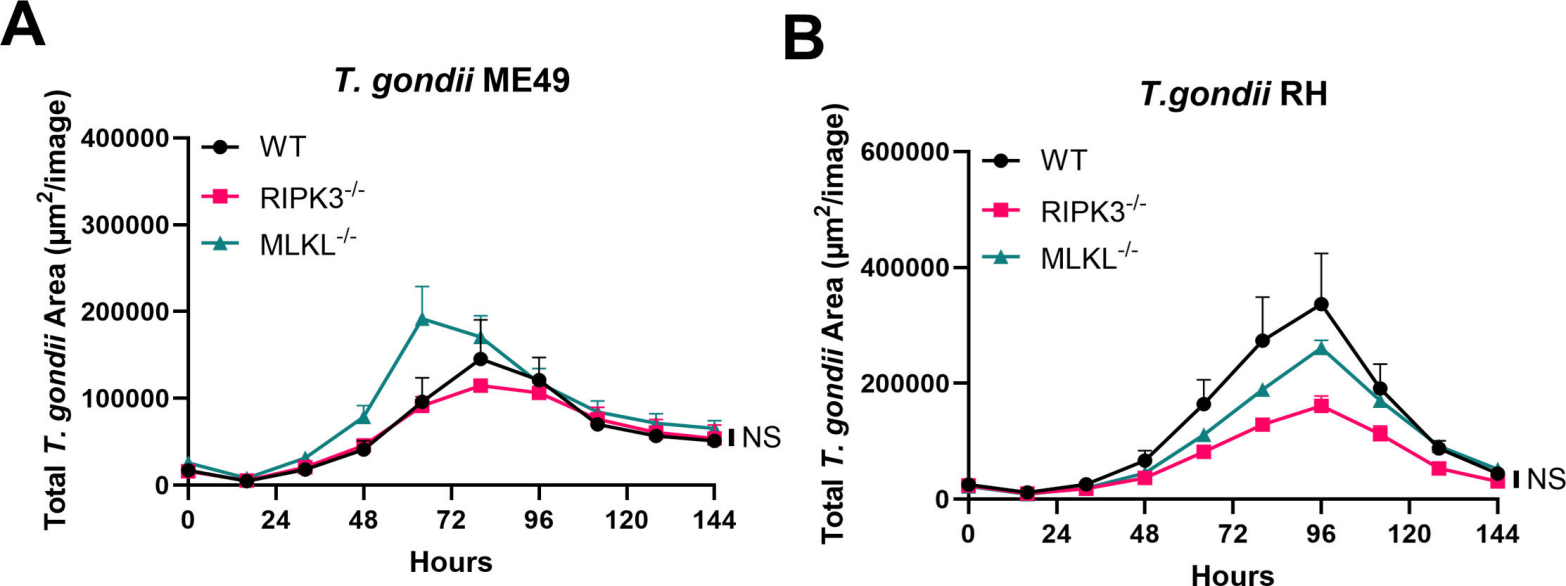
*T. gondii* growth is not affected by RIPK3 and MLKL deletions in naïve BMDMs. A total of 2 × 10^4^ BMDMs were seeded in a 96-well plate and infected with 2 × 10^4^ me49 mCherry tachyzoites or 1 × 10^4^ RH mCherry. (**A**) Total area of fluorescence signal from *T. gondii* at the indicated time points after WT, RIPK3^−/−^, and MLKL^−/−^ BMDMs were infected with mCherry ME49 *T. gondii*. Mean ± SEM (*n* = 4 independent samples). Two independent experiments. (**B**) Total fluorescence signal from *T. gondii* at the indicated time points after WT, RIPK3^−/−^, and MLKL^−/−^ BMDMs were infected with mCherry RH *T. gondii*. Mean ± SEM (*n* = 4 independent samples). Two independent experiments. (**A and B**) Statistical analysis was performed using a one-way ANOVA followed by post hoc Dunnett’s test comparing the area under the curve of WT to RIPK3^−/−^ or WT to MLKL^−/−^. * Indicates *P* < 0.05, ** indicates *P* < 0.01, *** indicates *P* < 0.001, and NS indicates not significant.

Given that RIPK3 is a well-established regulator of the necroptosis pathway (32), we investigated whether its absence would enhance *T. gondii* replication. To assess this, we used MLKL null (MLKL^−/−^) bone marrow-derived macrophages (BMDMs) as a control for RIPK3-dependent necroptosis. First, parasite replication was measured in naïve WT, RIPK3^−/−^, and MLKL^−/−^ BMDM following infection with mCherry Type II *T. gondii* (ME49) tachyzoites. The number of parasites per vacuole was manually quantified using microscopy, and no substantial differences were observed between the three genotypes during the initial 48 hours of infection (Fig. S1). To extend our analysis beyond 48 hours, we measured mCherry tachyzoite fluorescence every 16 hours up to 144 hours, revealing that *T. gondii* replication remained unaffected by the absence of RIPK3 or MLKL (Fig. 1A). Because different strain types of *T. gondii* elicit distinct host responses, including variations in cell death pathways (33), we compared the replication contrast of Type I (RH) and Type II (ME49). Infection with mCherry RH or ME49 *T. gondii* both showed no significant differences in replication among the genotypes in naïve BMDMs (Fig. 1B).

### TNF-α and Z-VAD-FMK limit *T. gondii* replication in a RIPK3- and MLKL-dependent manner

**Fig 2:**
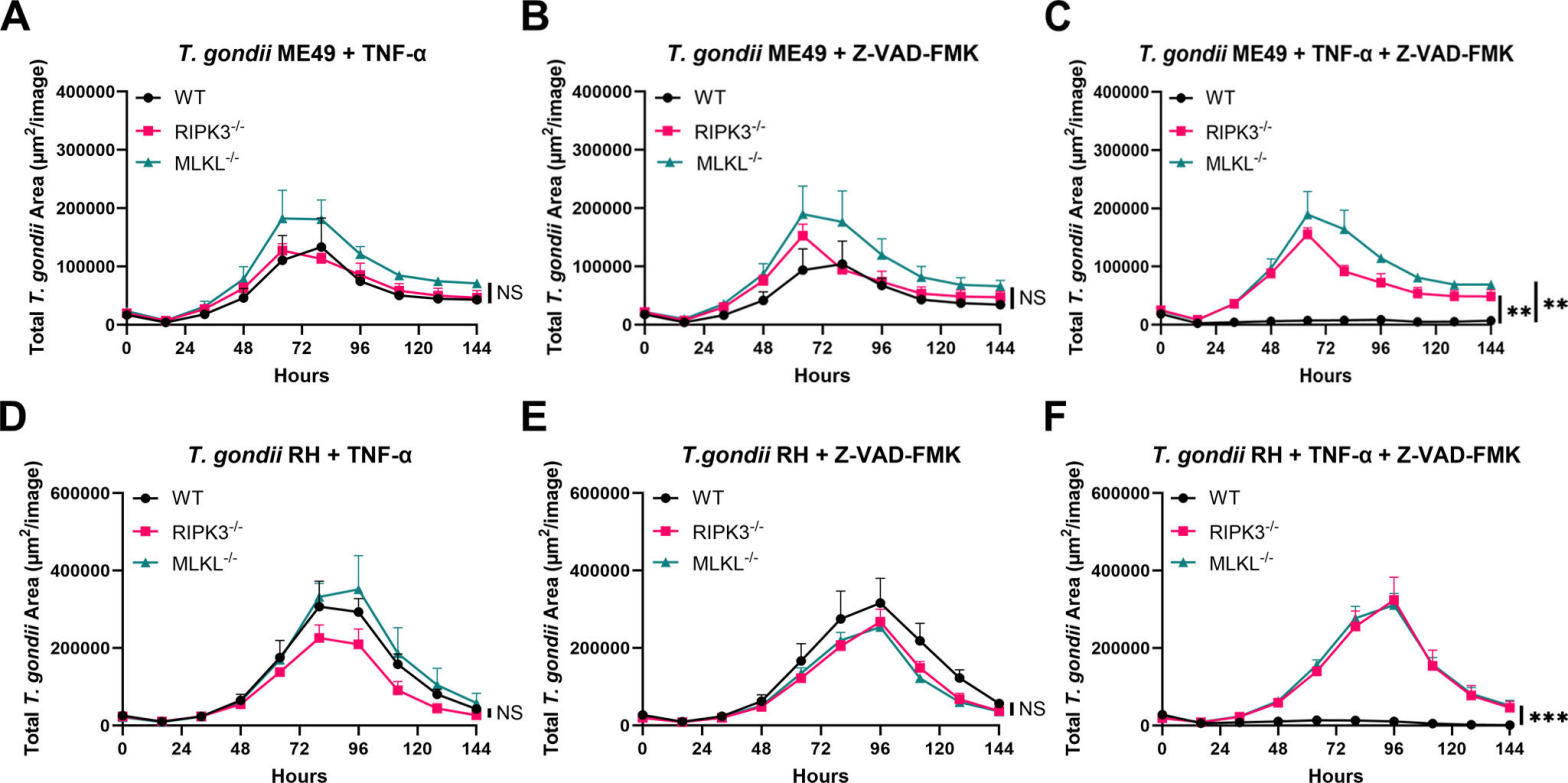
Both TNF-α and Z-VAD-FMK are necessary to trigger a host cell response that restricts *T. gondii* in BMDMs. A total of 2 × 10^4^ BMDMs were seeded in a 96-well plate and infected with 2 × 10^4^ me49 mCherry tachyzoites or 1 × 10^4^ RH mCherry. Reagents concentration: TNF-α (30 ng/mL) and Z-VAD(OH)-FMK (20 mM). (**A–C**) Total area of fluorescence signal from *T. gondii* at the indicated time points after WT, RIPK3^−/−^, and MLKL^−/−^ BMDMs were infected with mCherry ME49 *T. gondii*. Mean ± SEM (*n* = 4 independent samples). Two independent experiments. (**D–F**) Total area of fluorescence signal from *T. gondii* at the indicated time points after WT, RIPK3^−/−^, and MLKL^−/−^ BMDMs were infected with mCherry RH *T. gondii*. Mean ± SEM (*n* = 4 independent samples). Two independent experiments. (**A–F**) Statistical analysis was performed using a one-way ANOVA followed by post hoc Dunnett’s test comparing the area under the curve of WT to RIPK3^−/−^ or WT to MLKL^−/−^. * Indicates *P* < 0.05, ** indicates *P* < 0.01, *** indicates *P* < 0.001, and NS indicates not significant.

We examined whether activating the necroptosis pathway using the pro-inflammatory cytokine TNF-α was sufficient to induce necroptosis and affect *T. gondii* replication and survival in the absence of RIPK3 or MLKL in BMDMs. Our results demonstrated that TNF-α alone does not interfere with *T. gondii* pathogenesis, regardless of RIPK3 and MLKL status (Fig. 2A). To further evaluate the role of necroptosis, we pursued the same experiment, but this time, we added Z-VAD-FMK, a pan-caspase inhibitor that will block apoptosis and facilitate necroptosis by shifting the cell death response toward the necroptotic pathway (Fig. S2). However, Z-VAD-FMK alone also failed to affect *T. gondii* replication or survival, even without RIPK3 and MLKL (Fig. 2B). Given that TNF-α can trigger the necroptosis signaling cascade and Z-VAD-FMK prevents apoptosis, their combined use should effectively promote necroptosis. Our results demonstrated that when both TNF-α and Z-VAD-FMK were present, *T. gondii* replication was significantly reduced in BMDMs expressing RIPK3 and MLKL (Fig. 2C). Representative microscopic images of BMDMs treated with TNF-α and Z-VAD-FMK are shown in Fig. S3, demonstrating the decreased parasite burden in wild-type (WT) cells. In contrast, this effect was not observed in RIPK3 or MLKL-deficient BMDMs (Fig. 2C). We conducted this experiment using isolated peritoneal leukocytes, which also showed that when both TNF-α and Z-VAD-FMK were present, *T. gondii* replication was significantly reduced (Fig. S4). These results indicate that the suppression of *T. gondii* replication is contingent upon an intact necroptotic signaling axis, reinforcing the requirement of both RIPK3 and MLKL for effective parasite restriction. Similar results were seen for both Type I (Fig. 2D through F) and Type II (Fig. 2A through C) strains.

### RIPK1 inhibition prevents TNF-α and Z-VAD-FMK-mediated suppression of *T. gondii*

**Fig 3:**
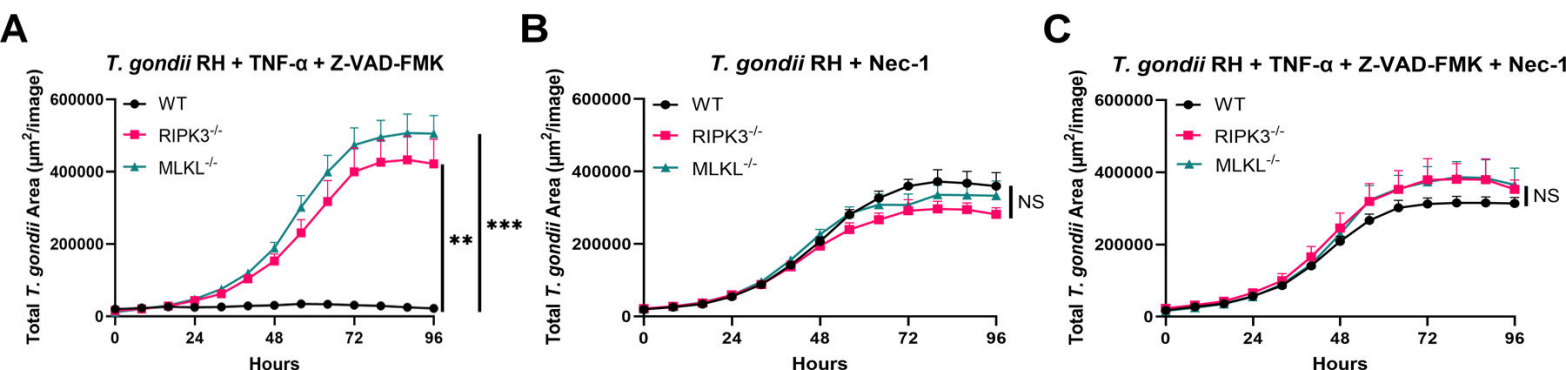
Necrostatin-1 prevents TNF-α and Z-VAD-FMK-mediated suppression of *T. gondii*. A total of 2 × 10^4^ BMDMs were seeded in a 96-well plate and infected with 1 × 10^4^ RH mCherry tachyzoites. Reagents concentration: TNF-α (30 ng/mL), Z-VAD(OH)-FMK (20 mM), and Necrostatin-1 (50 mM). (**A–C**) Total area of fluorescence signal from *T. gondii* at the indicated time points after WT, RIPK3^−/−^, and MLKL^−/−^ BMDMs were infected with mCherry RH *T. gondii*. Mean ± SEM (*n* = 3 independent samples). Two independent experiments. (**A–C**) Statistical analysis was performed using a one-way ANOVA followed by post hoc Dunnett’s test comparing the area under the curve of WT to RIPK3^−/−^ or WT to MLKL^−/−^. * Indicates *P* < 0.05, ** indicates *P* < 0.01, *** indicates *P* < 0.001, and NS indicates not significant.

To understand whether the complete execution of necroptosis or a Casp-8-independent mechanism was responsible for affecting *T. gondii* survival, we incorporated Nec-1, which will block the formation of the necrosome complex and the execution of the necroptotic pathway. As expected, when BMDMs were infected with *T. gondii* in the presence of Nec-1, parasite survival remained unaffected, even in cells expressing RIPK3 and MLKL (Fig. 3B). Based on these results, we examined the effect of Nec-1 when combined with TNF-α and Z-VAD-FMK to evaluate whether blocking RIPK1 signaling would rescue *T. gondii* replication suppression induced by these factors. When combined, parasite replication was unaffected under these conditions. Confirming that the suppression of *T. gondii* replication observed with TNF-α and Z-VAD-FMK requires functional RIPK1 signaling (Fig. 3C). In contrast, when TNF-α and Z-VAD-FMK were used without Nec-1 in the presence of RIPK3 and MLKL, *T. gondii* replication was significantly reduced (Fig. 3A), as we have seen before.

### Necroptosis induced by TNF-α and Z-VAD-FMK leads to *T. gondii* suppression in BMDMs

**Fig 4:**
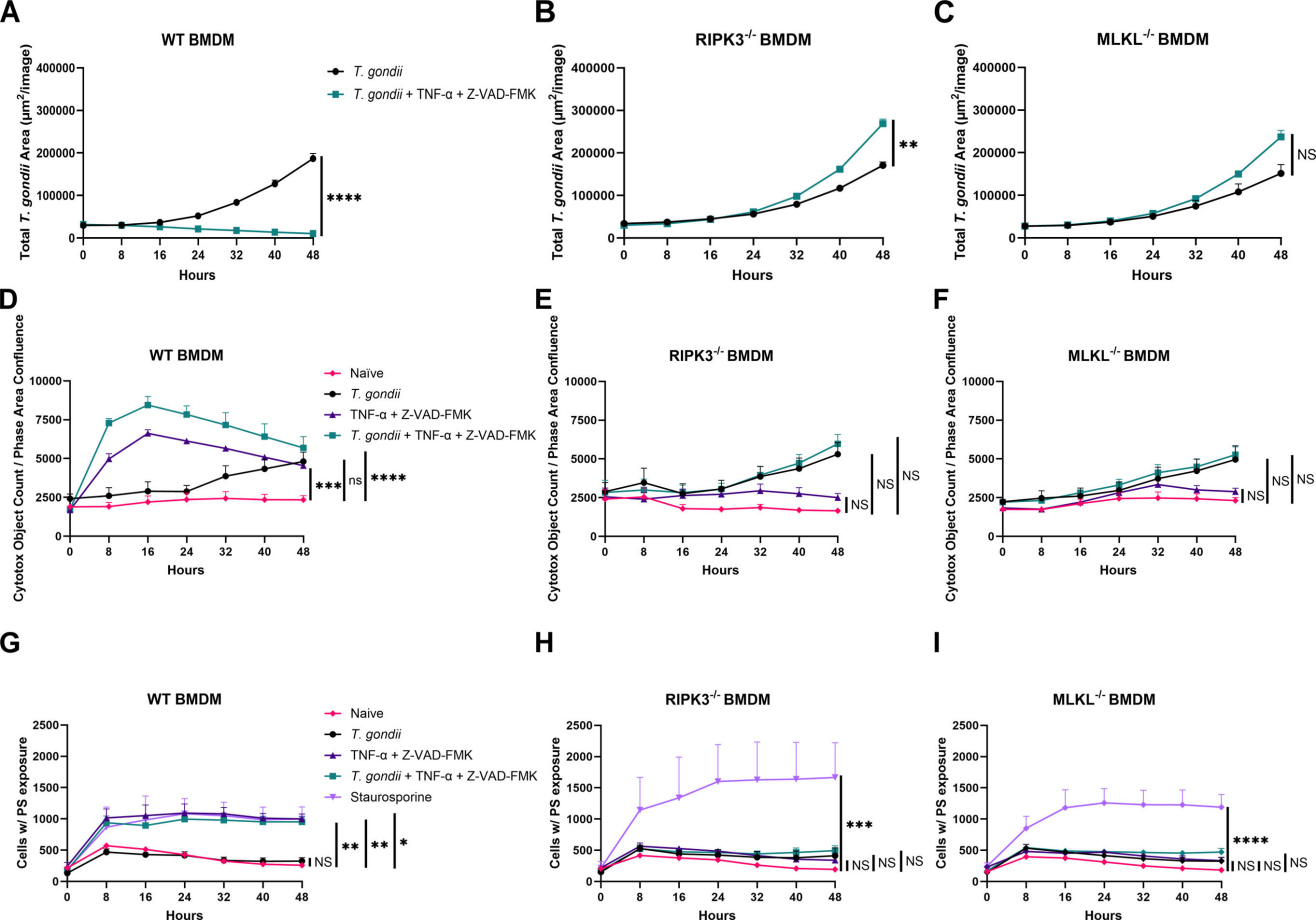
TNF-α and Z-VAD-FMK inhibit *T. gondii* growth by inducing host cell necroptosis. A total of 2 × 10^4^ BMDMs were seeded in a 96-well plate and infected with 1 × 10^4^ RH mCherry tachyzoites. Reagents concentration: TNF-α (30 ng/mL), Z-VAD(OH)-FMK (20 mM), Staurosporine (1 mM) independently, and together. InuCyte Cytotox Dye for Counting Dead Cells (Sartorius-4633) was added to measure cell membrane integrity disruption. For apoptosis/necroptosis quantification, Incucyte Annexin Dye for Apoptosis (Sartorius-4642) was added. (**A–C**) Total area of fluorescence signal from *T. gondii* at the indicated time points after WT, RIPK3^−/−^, and MLKL^−/−^ BMDMs were infected with mCherry RH *T. gondii*. Mean ± SEM (*n* = 6 independent samples). Two independent experiments. (**D–F**) Cytotox object count normalized to phase area confluence at indicated time points after WT, RIPK3^−/−^, and MLKL^−/−^ BMDMs were infected with mCherry RH *T. gondii*. Mean ± SEM (*n* = 6 independent samples). Two independent experiments. (**G–I**) Cells with phosphatidylserine (PS) exposure at indicated time points after WT, RIPK3^−/−^, and MLKL^−/−^ BMDMs were infected with mCherry RH *T. gondii*. Mean ± SEM (*n* = 6 independent samples). Two independent experiments. (**A–C**) Statistical analysis was performed using an unpaired *t*-test comparing the area under the curve of WT to RIPK3^−/−^ or WT to MLKL^−/−^. (**D–I**) Statistical analysis was performed using a one-way ANOVA followed by post hoc Dunnett’s test comparing the area under the curve of WT to RIPK3^−/−^ or WT to MLKL^−/−^. * Indicates *P* < 0.05, ** indicates *P* < 0.01, *** indicates *P* < 0.001, and NS indicates not significant.

To confirm that the reduction of parasitemia with TNF-α and Z-VAD-FMK in the presence of RIPK3 and MLKL was due to necroptosis, we assessed host cell survival during infection by measuring cell membrane integrity. When cell death is normalized to phase area confluency (Fig. 4D through F), WT BMDMs infected with *T. gondii* alone began to exhibit signs of membrane integrity disruption around 24 hours post-infection (Fig. 4D), consistent with parasite egress following replication. However, WT BMDMs exposed to *T. gondii* in the presence of TNF-α and Z-VAD-FMK showed significant disruption of cell membrane integrity as early as 8 hours post-infection when data are normalized to phase area confluency (Fig. 4D). In contrast, when the same conditions were applied to RIPK3^−/−^ and MLKL^−/−^ BMDMs, the only membrane disruption observed corresponded to the normal parasite egress, with no additional loss of integrity (Fig. 4E and F). Importantly, parasites that egress or are released by lysis of the host cell (8−16 hours post-infection) retained viability, suggesting that the observed phenotype reflects host cell death rather than direct parasite killing (Fig. S5). These findings suggest that the reduced parasitemia in WT BMDMs treated with TNF-α and Z-VAD-FMK is associated with a distinct form of cell death characterized by early, extensive membrane integrity disruption.

To further validate that this observed cell death was certainly necroptosis, we measured phosphatidylserine (PS) exposure and used the apoptosis inducer Staurosporine (STS) as a positive control. WT BMDMs treated with TNF-α and Z-VAD-FMK displayed PS exposure levels comparable to the STS control (Fig. 4G). In contrast, in RIPK3^−/−^ and MLKL^−/−-^ BMDMs, a significant PS signal was only observed in the STS-treated group, whereas TNF-α and Z-VAD-FMK treatment failed to elicit the same response (Fig. 4H and I). These combined results confirm that the reduction in *T. gondii* parasitemia observed in WT BMDMs treated with TNF-α and Z-VAD-FMK is a direct consequence of the PCD pathway dependent on RIPK3 and MLKL, necroptosis.

## DISCUSSION

Host resistance to intracellular pathogens like *T. gondii* depends on the precise coordination of innate immune signaling and other approaches, such as PCD. While apoptosis is a well-established form of PCD, necroptosis has emerged as a pro-inflammatory alternative crucial in limiting pathogen replication and driving the immune response (34). Our laboratory previously demonstrated that RIPK3 activity contributes to host susceptibility to oral *T. gondii* infection independently of MLKL and pattern recognition receptor Z-DNA binding protein 1 (ZBP1). ZBP1, despite being a known necroptosis initiator, does not confer protective immunity in this context (30). These findings suggest that RIPK3 may function beyond its classical necroptotic role, highlighting the need to further dissect the dynamics of PCD in host–parasite interactions. Another study found that the bradyzoite stage of *T. gondii* secretes the effector TgNSM, which cooperates with TgIST to suppress IFN-driven expression of key necroptosis mediators, including PKR and MLKL. This dual-effector strategy prevents host cell necroptotic death, thereby preserving the parasite’s intracellular niche during chronic infection (31). However, it remains unresolved whether *T. gondii* can effectively suppress necroptosis across different stages of infection or in various host cell types.

Here, we determined whether activation of necroptosis could serve as a host defense strategy to limit *T. gondii* replication, particularly through the TNF-α-driven necroptotic axis (22), and to identify whether this mechanism depends on key mediators such as RIPK3 and MLKL. Our findings demonstrate that the induction of the necroptosis pathway can significantly restrict *T. gondii* replication in BMDMs, but only under specific conditions. We show that combining TNF-α and the pan-caspase inhibitor Z-VAD-FMK significantly reduces *T. gondii* parasitemia in WT BMDMs, but not in BMDMs deficient in RIPK3 or MLKL. This suppression of parasite replication was due to the successful completion of the necroptosis pathway, which eliminated the BMDMs, leading to the absence of host cells required for survival and replication of *T. gondii*. To contextualize the physiological importance of our exogenous TNF-α treatment, we quantified endogenous TNF-α production by BMDMs during *T. gondii* infection and found it to be significantly lower than the levels used in our experiments, supporting the reason for exogenous supplementation (Fig. S6).

We tested both Type I (RH) and Type II (ME49), and despite their differences in virulence, both Type I and Type II strains of *T. gondii* showed reduced parasitemia in wild-type BMDMs treated with TNF-α and the pan-caspase inhibitor Z-VAD-FMK. This effect was absent in BMDMs lacking RIPK3 or MLKL. This indicates that the necroptotic pathway can serve as a broad-spectrum defense mechanism when fully inducing necroptosis in BMDMs. These data underscore the requirement of a functional necroptotic signaling axis to limit *T. gondii* replication and highlight necroptosis as a potential mechanism of host resistance against intracellular pathogens.

We found that in the absence of any necroptosis stimuli, deletion of RIPK3 or MLKL did not impact *T. gondii* replication in BMDMs (Fig. 1). This result was confirmed through both early-stage manual quantification (Fig. S1) and long-term kinetic analysis (Fig. 1) using mCherry fluorescence. Even across strains with different virulence profiles, such as RH and ME49, the replication kinetics remained unchanged in WT, RIPK3^−/−^, and MLKL^−/−^ BMDMs. After 72−96 hours, we started to observe a decrease in parasite signal, indicating a lack of new host cells to invade, which leads to parasite death. Our findings suggest that, under basal conditions, *T. gondii* is capable of replicating efficiently in macrophages regardless of the presence or absence of key necroptotic mediators. Therefore, under our conditions, necroptosis does not appear to be spontaneously activated in BMDMs during *T. gondii* RH or ME49 infection, and its role in host defense must be induced through additional signals. Unlike other intracellular pathogens such as *Salmonella typhimurium* (35), *Staphylococcus aureus* (36), and influenza A virus (37), which can independently trigger necroptosis.

To probe the conditions necessary to activate necroptosis, we evaluated the impact of TNF-α, a well-known inducer of necroptosis (22, 38), and Z-VAD-FMK, a pan-caspase inhibitor used to sensitize cells to inducers of necroptosis (39, 40). When applied independently, neither TNF-α nor Z-VAD-FMK influenced *T. gondii* replication in WT, RIPK3^−/−^, and MLKL^−/−^ BMDMs, indicating that these agents alone are insufficient to trigger a host defense response that reduces parasite survival or replication (Fig. 2A, B, D and E). However, co-treatment with both TNF-α and Z-VAD-FMK resulted in the parasite’s inability to replicate, but only in WT BMDMs (Fig. 2C and F). This inability to replicate was absent in RIPK3^−/−^ and MLKL^−/^ BMDMs (Fig. S3), indicating that the observed anti-parasitic effect is contingent upon intact necroptotic machinery. These data suggest that both upstream (TNF-α) and downstream (Z-VAD-FMK) components are necessary to induce a host response that can limit *T. gondii* replication in BMDMs.

Further evidence supporting the central role of necroptosis came from using Nec-1, a selective inhibitor of RIPK1 and a well-known necroptosis inhibitor (29, 41). When Nec-1 was added to BMDMs treated with TNF-α and Z-VAD-FMK, the suppression of *T. gondii* replication was reversed entirely, confirming that RIPK1 activity is essential for initiating necroptotic signaling (Fig. 3). Notably, Nec-1 alone did not impact parasite replication, suggesting that the drug does not interfere with parasite viability or host basal immunity but specifically inhibits the necroptosis pathway (Fig. 3C). These results solidify the conclusion that the full engagement of RIPK1, RIPK3, and MLKL is required to achieve necroptosis-mediated restriction of *T. gondii* in BMDMs.

To confirm that the reduction in parasite load resulted from successful complete necroptosis, we assessed markers of PCD, including membrane integrity disruption and PS exposure. In healthy cells, PS is confined to the inner leaflet of the plasma membrane; however, upon receiving apoptotic stimuli, PS translocates to the outer surface, allowing for measurable binding. In necrotic cells, due to membrane integrity disruption, PS is also accessible for binding, although this occurs through the binding reagent gaining access to the intracellular area of the cell. To distinguish these differences, we used Staurosporine, a well-established apoptosis inducer, as a positive control and our necroptosis controls, RIPK^−/−^ and MLKL^−/−^ BMDMs (Fig. 4G through I). WT BMDMs treated with TNF-α and Z-VAD-FMK displayed significant early membrane disruption (Fig. 4D through F) and PS exposure (Fig. 4G through I), indicative of necroptotic cell death. In contrast, RIPK3^−/−^ and MLKL^−/−^ BMDMs did not exhibit these markers under the same treatment, further supporting that necroptosis was not engaged in the absence of these proteins. These functional assays validate that the anti-parasitic effect is directly associated with RIPK3- and MLKL-dependent cell death, establishing necroptosis as a potent host strategy to eliminate *T. gondii*-infected cells and restrict parasite propagation. While not the focus of this study, it is worth noting that Z-VAD-FMK, the pan-caspase inhibitor used herein, also blocks a third well-known mechanism of programed cell death, the inflammasome activation, which may be relevant to the observed phenotypes. Future studies will compare the fate of parasites after necroptosis (TNFα+ Z-VAD-FMK) with that of other types of programed cell death, such as FasL-induced apoptosis or LPS + nigericin-induced pyroptosis, to determine whether *T. gondii* viability varies across different host cell death pathways.

## MATERIALS AND METHODS

### Mice for all experiments

All mice used were from the C57BL/6 background. WT mice were originally purchased from JAX but have been bred in the UW-Madison vivarium with all the other strains used for these studies. RIPK3 null (RIPK3^−/−^) mice were provided from Genentech (42). MLKL null (MLKL^−/−^) mice were provided by Doug Green at St. Jude Children’s Research Hospital (43). The RIPK3^−/−^ strain was genotyped by PCR with three primers: 5′-AGAAGATGCAGCAGCCTCAGCT, 5′-ACGGACCCAGGCTGACTTATCTC, and 5′-GGCACGTGCACAGGAAATAGC. The MLKL^−/−^ strain was also genotyped by PCR with three primers: 5′-TATGACCATGGCAACTCACG, 5′-ACCATCTCCCCAAACTGTGA, and 5′-TCCTTCCAGCACCTCGTAAT.

### Parasites and host cell culture

The ME49 mCherry (30) and RH mCherry *T. gondii* strains were used for all experiments and maintained in human foreskin fibroblast (HFF) cells in a humidified 37°C incubator with 5% CO_2_. HFFs were cultured in Dulbecco’s modified Eagle Medium (Gibco) supplemented with 10% fetal bovine serum, 2 mM L-glutamine or GlutaMAX, 10 mM HEPES, and 1% penicillin-streptomycin. BMDMs were harvested from 8- to 10-week-old mice and grown in 20% L929-conditioned RPMI 1640 medium as described previously (44).

### *In vitro* manual parasite quantification

The parasite burden *in vitro* was manually quantified by immunofluorescence in BMDMs. A total of 1 × 10^5^ viable BMDMs were seeded on glass coverslips and infected with me49 mCherry 1 × 10^5^ mCherry tachyzoites. At 3 hours post-infection, the medium was changed to remove extracellular parasites. At 48 hours post-infection, parasites per vacuole were counted. Each condition was performed on triplicate coverslips, and the experiment was repeated, providing a total of six coverslips per condition. mCherry fluorescence was preserved using Vectashield antifade mounting medium with 4′,6-diamidino-2-phenylindole (H1500; Vector Laboratories) and by photographing 10 random fields per coverslip with a Zeiss Axioplan III motorized microscope with a 40× oil objective. All photographs were blinded before counting. All parasites per vacuole were counted for every vacuole in the photograph, with at least 260 vacuoles counted per condition.

### *In vitro* kinetic parasite quantification

The parasite burden *in vitro* kinetically was determined by immunofluorescence in BMDMs. A total of 2 × 10^4^ BMDMs or peritoneal leukocytes were seeded in a 96-well plate and infected with 2 × 10^4^ me49 mCherry tachyzoites or 1 × 10^4^ RH mCherry. At 3 hours post-infection, the medium was changed to remove extracellular parasites, and BMDMs were treated with TNF-α (30 ng/mL; Peprotech-315-01A), Z-VAD(OH)-FMK (20 mM; SC-311560), Necrostatin-1 (50 mM; SC-200142), and Staurosporine (1 mM; S6942) independently and together. InuCyte Cytotox Dye for Counting Dead Cells (Sartorius-4633) was added to measure cell membrane integrity disruption. For apoptosis/necroptosis quantification, Incucyte Annexin Dye for Apoptosis (Sartorius-4642) was added. Plates were placed for imaging on an IncuCyte Live Cell Analysis system (Sartorius) and imaged every 8−16 hours. The IncuCyte was housed in a humidified 37°C incubator with 5% CO_2_. The red channel used an acquisition time of 300 ms for all replicates and samples. All the conditions were analyzed in duplicate or triplicate, and the experiment was repeated. As a control, we considered a subset of the wells with BMDMs uninfected and/or unstimulated, which were processed and analyzed equally.

### Plaque assay

Plaque assays were performed by seeding HFF cells in 24-well plates until a confluent monolayer was formed. HFF cells were infected with the supernatant of an ongoing BMDM *T. gondii* RH mCherry infection. Supernatant was collected at 8 or 16 hours post-infection.

Plaque assay plates were left undisturbed for 7 days to allow the plaques to form. The medium was removed, and the cells were fixed with methanol and stained with crystal violet for 20 min. The samples were then rinsed with water, left to air dry overnight, and photographed. Plaques were counted using ImageJ software by analyzing the number of individual plaques formed.

### Induced peritoneal leukocyte collection

Each mouse was injected with 5 mL thioglycollate medium into the peritoneal cavity. After 3−5 days, mice were humanely euthanized and cleaned with 70% alcohol. A small incision was made to expose the abdominal wall without cutting into the cavity. Cold phosphate-buffered saline was injected into the peritoneal cavity, massaged, and then collected. The collected cell suspension was spun at 1,500 RPM for 8 minutes, the supernatant was discarded, and the cells were resuspended in media for counting.

### TNF-α bead assay

*In vitro* cytokine levels were measured using the BD cytometric bead array mouse inflammation kit (BD Biosciences). Media supernatant was collected from BMDM *T. gondii* RH mCherry infection at 72 hours post-infection. Samples were processed according to the manufacturer’s instructions and analyzed using an Attune flow cytometer (Thermo-Fisher) at the University of Wisconsin Carbone Cancer Center. Further analysis was performed using the FlowJo software.

## SUPPLEMENTAL MATERIAL

Fig. S1 iai.00479-25-s0001.tif *T. gondii* growth rate not affected by RIPK3 and MLKL deletions in naïve BMDMs.

Fig. S2 iai.00479-25-s0002.png Modulation of programed cell death through TNFR1 signaling.

Fig. S3 iai.00479-25-s0003.png Images of *T. gondii* growth in BMDMs treated with TNF-α and Z-VAD at 48 hours post-infection.

Fig. S4 iai.00479-25-s0004.tif TNF-α and Z-VAD-FMK trigger a host cell response that restricts *T. gondii* in isolated peritoneal leukocytes.

Fig. S5 iai.00479-25-s0005.tif Plaque assay from supernatant of BMDM and *T. gondii* infection.

Fig. S6 iai.00479-25-s0006.tif Endogenous TNF-α levels in BMDMs in response to *T. gondii* and reagents.

Supplemental material iai.00479-25-s0007.docx Supplemental figure legends.

ASM does not own the copyrights to Supplemental Material that may be linked to, or accessed through, an article. The authors have granted ASM a non-exclusive, world-wide license to publish the Supplemental Material files. Please contact the corresponding author directly for reuse.
