# Supplementary figures and images for: Induced necroptosis limits Toxoplasma gondii replication in a RIPK3/MLKL-dependent manner
Source: Infect Immun. 2025 Oct 7;93(11):e00479-25. doi: 10.1128/iai.00479-25 (PMC12604491; doi:10.1128/iai.00479-25)

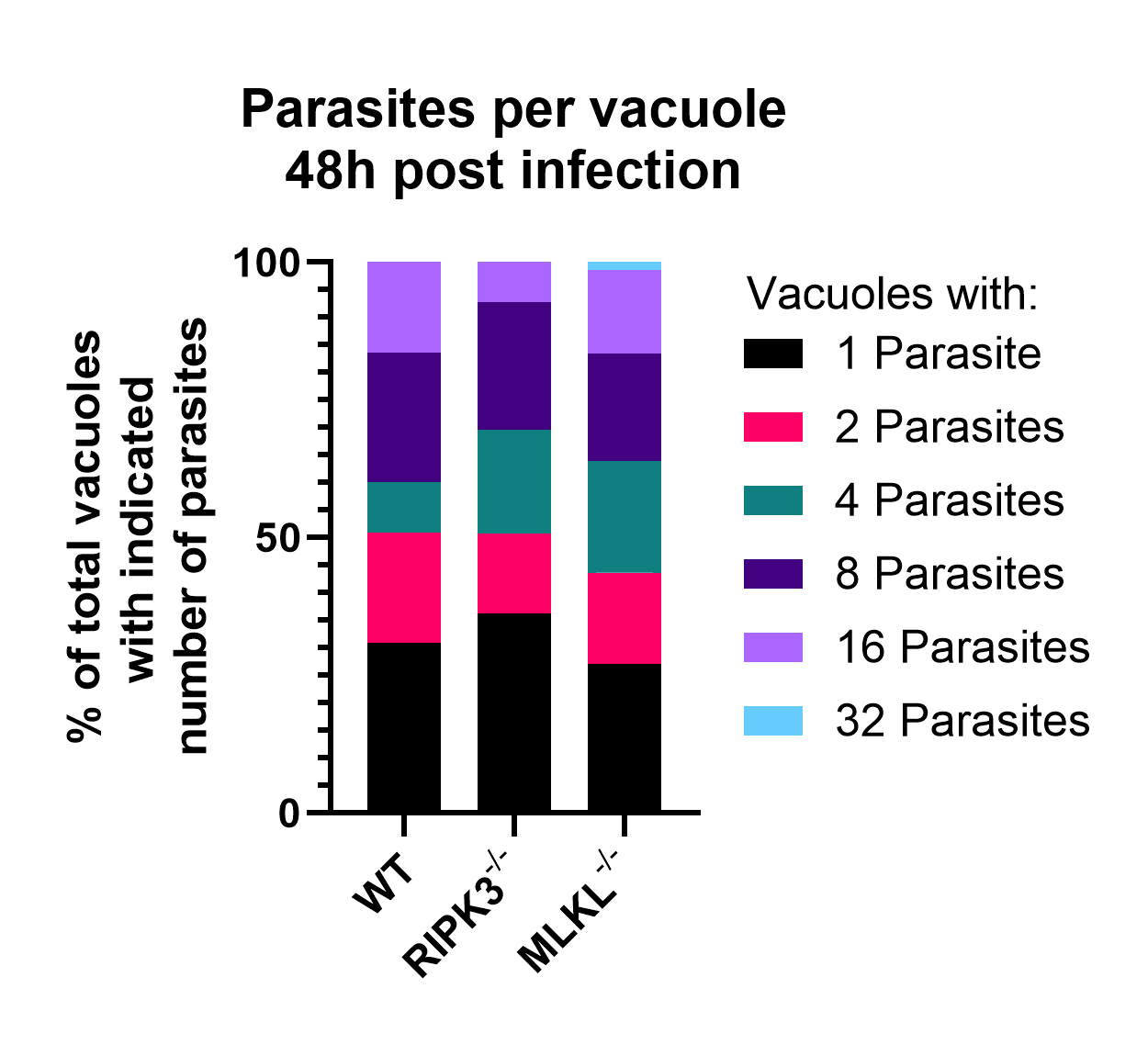

Supplement: Fig. S1 — T. gondii growth rate not affected by RIPK3 and MLKL deletions in naïve BMDMs. [file iai.00479-25-s0001.tif]

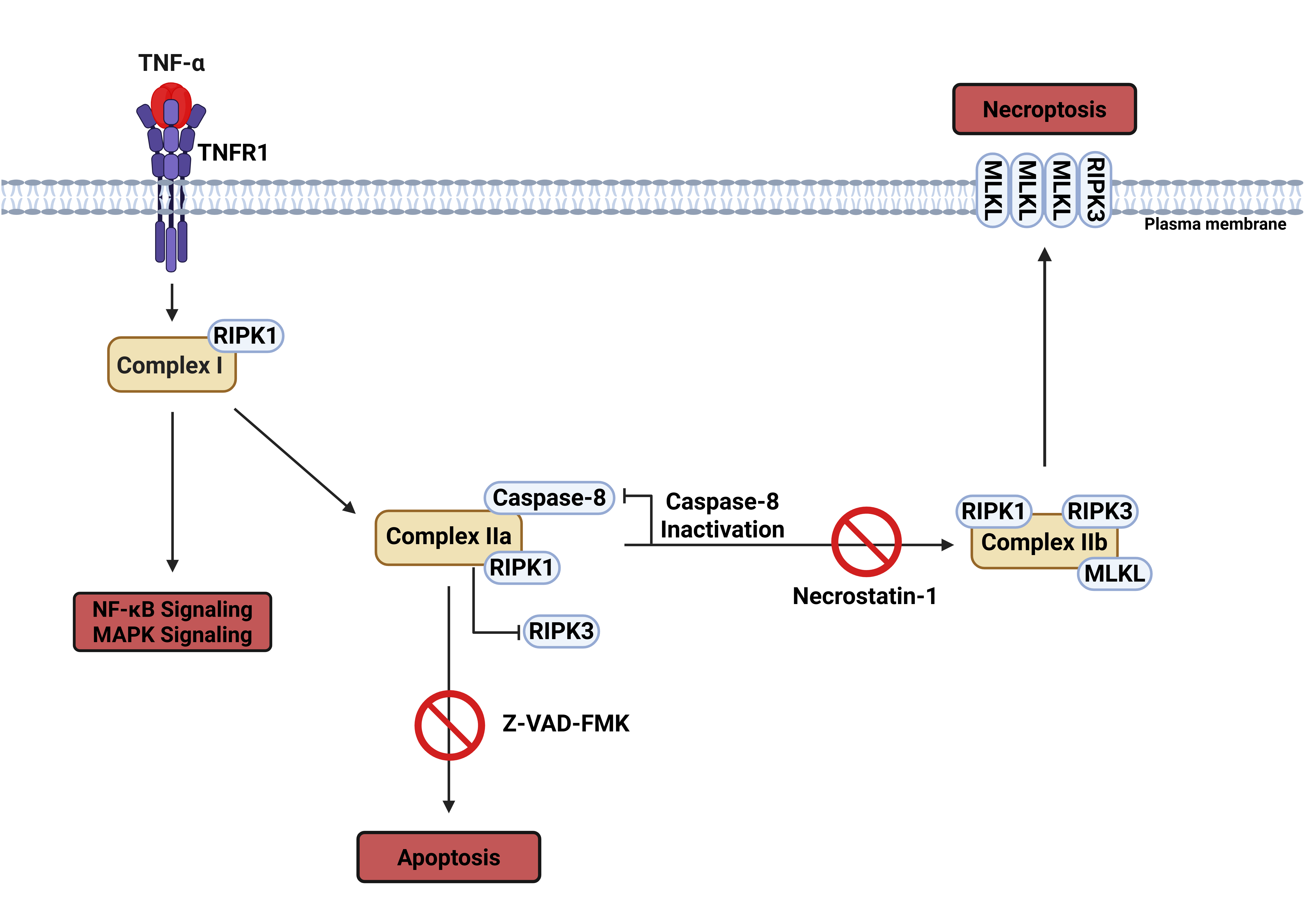

Supplement: Fig. S2 — Modulation of programed cell death through TNFR1 signaling. [file iai.00479-25-s0002.png]

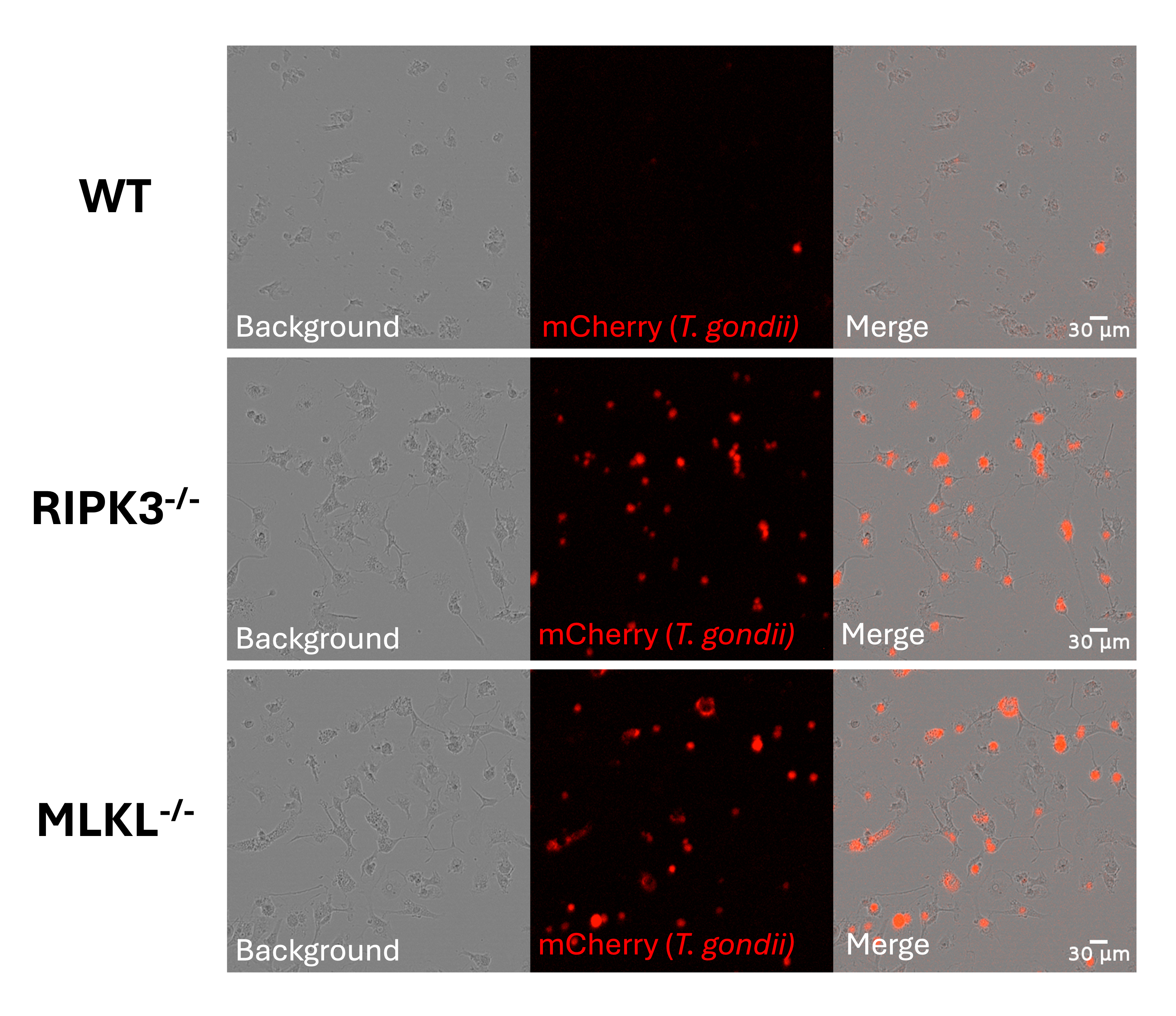

Supplement: Fig. S3 — Images of T. gondii growth in BMDMs treated with TNF-α and Z-VAD at 48 hours post-infection. [file iai.00479-25-s0003.png]

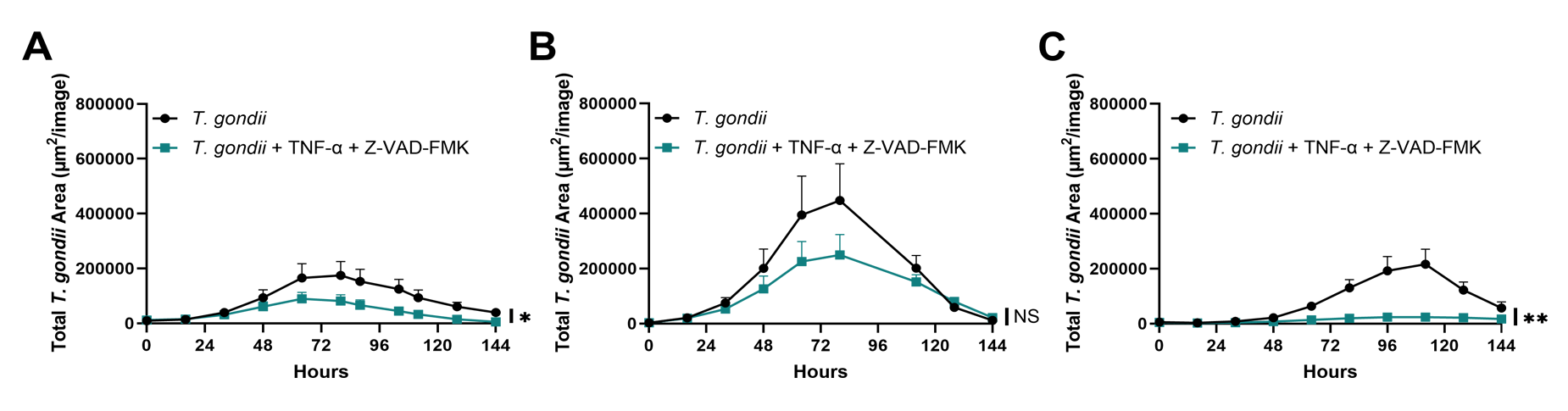

Supplement: Fig. S4 — TNF-α and Z-VAD-FMK trigger a host cell response that restricts T. gondii in isolated peritoneal leukocytes. [file iai.00479-25-s0004.tif]

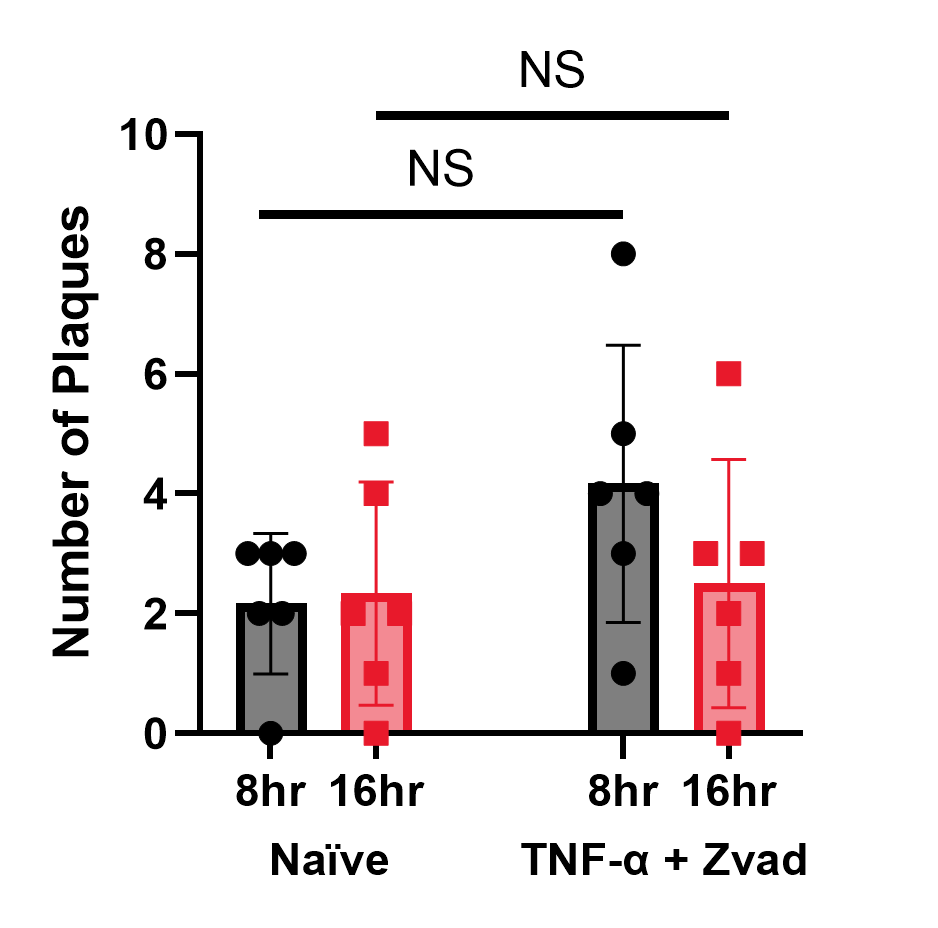

Supplement: Fig. S5 — Plaque assay from supernatant of BMDM and T. gondii infection. [file iai.00479-25-s0005.tif]

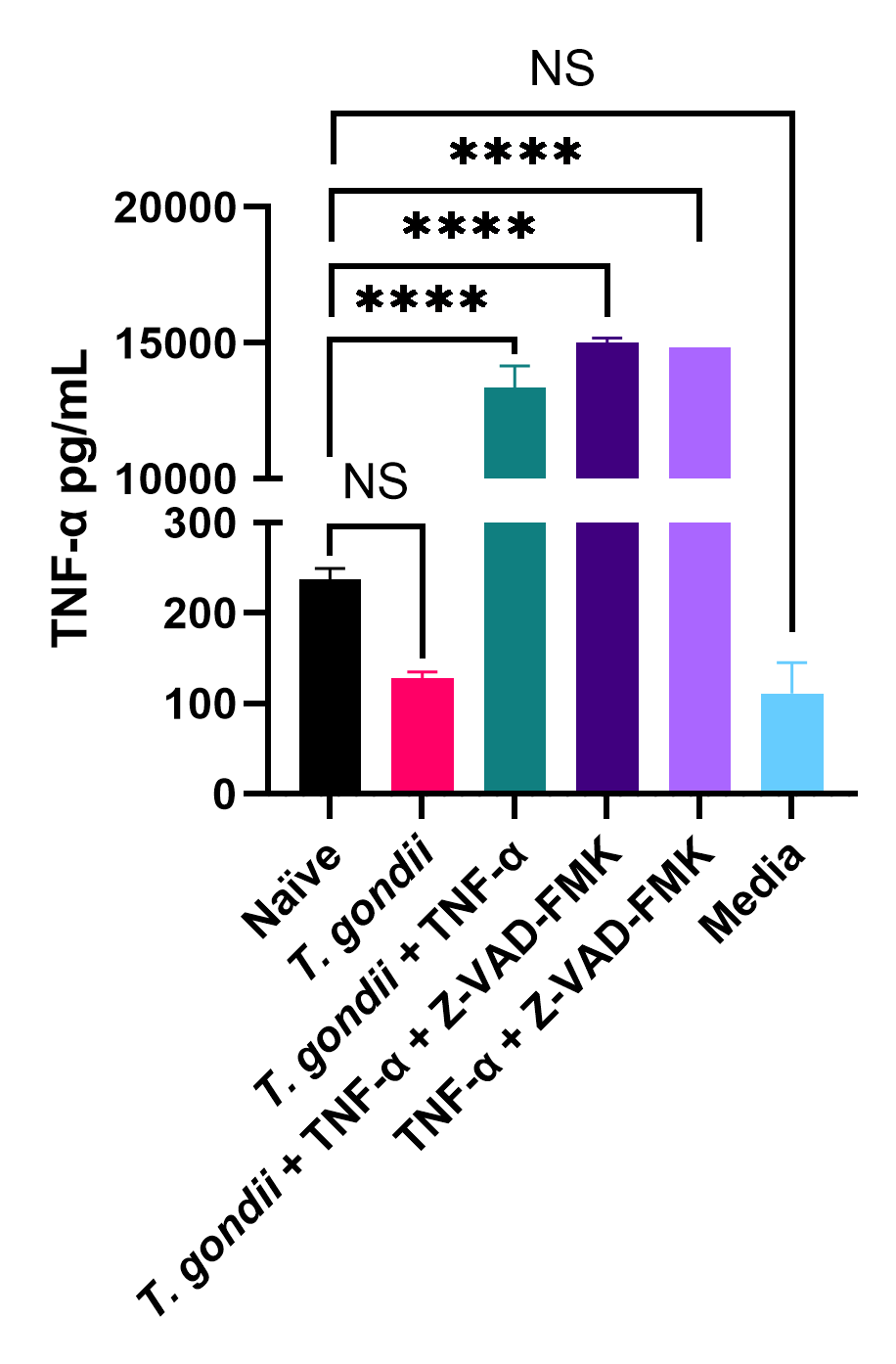

Supplement: Fig. S6 — Endogenous TNF-α levels in BMDMs in response to T. gondii and reagents. [file iai.00479-25-s0006.tif]
