# Supplementary material for: Induced necroptosis limits Toxoplasma gondii replication in a RIPK3/MLKL-dependent manner
Source: Infect Immun. 2025 Oct 7;93(11):e00479-25. doi: 10.1128/iai.00479-25 (PMC12604491; doi:10.1128/iai.00479-25)
Supplement: Supplemental material — Supplemental figure legends. [file iai.00479-25-s0007.docx]

# **Supplemental Figures Legends**

**Fig. S1: *T. gondii* growth rate not affected by RIPK3 and MLKL deletions in naïve BMDMs.** WT, RIPK3^-/-^ and MLKL^-/-^ BMDMs (1 × 10^5^ ) were infected with mCherry ME49 *T. gondii* (1 × 10^5^ ) for 48h, then fixed and incubated with primary antibody against *T. gondii* surface antigen 1 (SAG1) and counterstained with DAPI. Shown are representative immunofluorescence images of WT, RIPK3^-/-^ and MLKL^-/-^ BMDMs infected by ME49 *T. gondii*. Bottom: percentage of vacuoles with 1, 2, 4, 8, 16 or 32 parasites per vacuole, quantified from 10 random fields of view per coverslip. Each condition was performed on triplicate coverslips, and the experiment was repeated, providing a total of 6 coverslips per condition.

**Fig. S2: Modulation of programmed cell death through TNFR1 signaling.** Schematic illustrating the TNFR1 signaling pathway and its link to PCD, highlighting the use of the pan-caspase inhibitor Z-VAD-FMK and the RIPK1 inhibitor Nec-1.

**Fig. S3: Images of *T. gondii* growth in BMDMs treated with TNF-α and Z-VAD at 48 hours post-infection.** WT, RIPK3^-/-^, MLKL^-/-^ BMDMs infected with Type I RH in the presence of TNF-α and Z-VAD-FMK at 48h post-infection. Shown are representative IncuCyte images. A total of 2 x 10^4^ BMDMs were seeded in a 96-well plate and infected with 2 x 10^4^ me49 mCherry tachyzoites or 1 x 10^4^ RH mCherry. Reagents concentration: TNF-α (30ng/mL), Z-VAD(OH)-FMK (20mM).

**Fig. S4: TNF-α and Z-VAD-FMK trigger a host cell response that restricts *T. gondii* in isolated peritoneal leukocytes.** A total of 2 x 10^4^ isolated peritoneal leukocytes were seeded in a 96-well plate and infected with 1 x 10^4^ RH mCherry. Reagents concentration: TNF-α (30ng/mL), Z-VAD(OH)-FMK (20mM). Total area of fluorescence signal from *T. gondii* at the indicated time points after isolated peritoneal leukocytes were infected with mCherry RH T*. gondii*. Mean ± SEM (n=6 independent samples). Three independent experiments. Statistical analysis was performed using a one-way ANOVA followed by post hoc Dunnett’s test comparing the area under the curve of Naive to TNFα + Zvad-FMK * indicates p<0.05. ** indicates p<0.01. *** indicates p<0.001. NS indicates not significant.

**Fig. S5: Plaque assay from supernatant of BMDM and *T. gondii* infection.** Parasites in the supernatant on an ongoing infection of BMDM (2 x 10^4^ ) and *T. gondii* RH mCherry (1x10^4^) were collected at 8 and 16 hours post-infection. Plaque assay was performed in a confluent HFF monolayer and left undisturbed for 7 days. Plaques were then photographed and counted. (n=4 independent samples). Three independent experiments. Statistical analysis was performed using a unpaired two-tailed Student's t-test, * indicates p<0.05. ** indicates p<0.01. *** indicates p<0.001. NS indicates not significant.

**Fig. S6 Endogenous TNF-α Levels in BMDMs in Response to *T. gondii* and Reagents.** *In vitro* BMDMs (2x10^4^) cytokine levels were measured using the BD cytometric bead array mouse inflammation kit (BD Biosciences) after 72 hours postinfection with *T. gondii* RH mCherry (1x10^4^). Samples were processed using an Attune flow cytometer (Thermo-Fisher) and further analyzed with FlowJo software. (n=2 independent samples). One independent experiment. Statistical analysis was performed using a one-way ANOVA followed by post hoc Dunnett’s test comparing Naive against the others * indicates p<0.05. ** indicates p<0.01. *** indicates p<0.001. NS indicates not significant.
